# Multi-Objective Optimization in Air-to-Air Communication System Based on Multi-Agent Deep Reinforcement Learning

**DOI:** 10.3390/s23239541

**Published:** 2023-11-30

**Authors:** Shaofu Lin, Yingying Chen, Shuopeng Li

**Affiliations:** Faculty of Information Technology, Beijing University of Technology, Beijing 100124, China; linshaofu@bjut.edu.cn (S.L.); chenyingy@emails.bjut.edu.cn (Y.C.)

**Keywords:** unmanned aerial vehicle (UAV), mobile edge computing (MEC), wireless power transfer (WPT), multi-objective optimization (MOO), multi-agent deep reinforcement learning (MADRL)

## Abstract

With the advantages of real-time data processing and flexible deployment, unmanned aerial vehicle (UAV)-assisted mobile edge computing systems are widely used in both civil and military fields. However, due to limited energy, it is usually difficult for UAVs to stay in the air for long periods and to perform computational tasks. In this paper, we propose a full-duplex air-to-air communication system (A2ACS) model combining mobile edge computing and wireless power transfer technologies, aiming to effectively reduce the computational latency and energy consumption of UAVs, while ensuring that the UAVs do not interrupt the mission or leave the work area due to insufficient energy. In this system, UAVs collect energy from external air-edge energy servers (AEESs) to power onboard batteries and offload computational tasks to AEESs to reduce latency. To optimize the system’s performance and balance the four objectives, including the system throughput, the number of low-power alarms of UAVs, the total energy received by UAVs and the energy consumption of AEESs, we develop a multi-objective optimization framework. Considering that AEESs require rapid decision-making in a dynamic environment, an algorithm based on multi-agent deep deterministic policy gradient (MADDPG) is proposed, to optimize the AEESs’ service location and to control the power of energy transfer. While training, the agents learn the optimal policy given the optimization weight conditions. Furthermore, we adopt the K-means algorithm to determine the association between AEESs and UAVs to ensure fairness. Simulated experiment results show that the proposed MODDPG (multi-objective DDPG) algorithm has better performance than the baseline algorithms, such as the genetic algorithm and other deep reinforcement learning algorithms.

## 1. Introduction

### 1.1. Background and Related Works

As an emerging airborne platform, unmanned aerial vehicles (UAVs) have many advantages, such as low prices, high flexibility, and easy deployment. Therefore, they have been widely used in both civil and military fields, such as precision agriculture [1], building inspection [2], UAV swarm operation [3], and target detection [4]. Meanwhile, UAVs can be integrated into mobile edge computing (MEC) networks as a complement to the terrestrial communication infrastructure [5]. UAV-assisted edge computing systems can be deployed more flexibly than traditional edge computing systems. In addition, the line-of-sight channel model of UAVs can be utilized to improve the coverage and throughput of wireless communication networks [6].

Though UAV-assisted edge computing systems have many advantages and vast application prospects, they also face challenges. In UAV scenarios such as target striking, remote reconnaissance, wide-area surveillance, and combat support, the computational capability and energy resources of UAVs are in high demand. UAVs use batteries with limited capacity that cannot continuously provide energy. This restricts the range of applications for UAVs and their adaptability for computationally intensive application scenarios. Several energy replenishment strategies for UAVs have been proposed in existing works, including deploying ground-based fixed charging stations, utilizing vehicles as mobile charging stations [7], replacing batteries periodically, utilizing solar energy [8], and so on. However, these methods have limited effectiveness in improving the battery life of UAVs. UAVs frequently fly away from the work area to recharge, which not only interrupts the ongoing mission but also increases flight energy consumption.

In recent years, wireless power transfer (WPT) technology has been proposed and applied. WPT technology transmits energy wirelessly so that UAVs do not have to land or swap batteries. Therefore, it is considered as a promising solution to the UAV energy supply problem [9]. In UAV-supported WPT systems, UAVs can receive power stably and continuously over a wireless link and convert it into energy to improve the battery life [10]. Several studies have been devoted to building novel UAV-supported WPT architectures that utilize UAVs as mobile energy transmitters to power ground and airborne devices ([11,12,13,14]). Therefore, the UAV-assisted edge computing system, combined with WPT technology, can not only utilize the advantages of the UAV-assisted edge computing system, but also make up for the shortcomings of the limited battery capacity of the UAV. He et al. [15] proposed a resource allocation strategy for a UAV-assisted non-linear energy harvesting MEC system. Ground-based devices can power batteries by harvesting energy from UAVs and also offload computational tasks to UAVs to decrease communication latency. Liu et al. [16] proposed that, by equipping the UAVs with an energy transmitter and a MEC server, they can provide energy to the sensor devices and support their computational tasks. A joint optimization problem is constructed on this basis, involving CPU control and trajectory optimization. Xu et al. [17] designed a system that allowed Internet of Things (IoT) devices to similarly collect energy from the UAV and offload their computational tasks to the UAV, with the optimization goal of minimizing the energy consumption of the UAV. Yu et al. [18] proposed a UAV-assisted wireless powered IoT network. During hovering, the UAV operates in full-duplex mode and can simultaneously collect data from target devices and charge other devices within its coverage area. 

Most of the current research focuses on air-to-ground communication systems (A2GCS) for UAVs serving ground devices. In contrast, few existing works have been conducted on air-to-air communication systems (A2ACSs). An A2ACS allows the exchange and supplement of resources between UAVs. This type of resource sharing not only extends the battery life of UAVs, but also improves the reliability and anti-jamming ability of the whole system. However, existing research on A2ACSs primarily focuses on the management of a single category of resources of UAVs. Oubbati et al. [19] proposed to utilize a set of airborne energy stations to provide energy for UAVs, and effectively optimize the energy delivery efficiency of UAVs through deep reinforcement learning algorithms. This study did not consider UAV computing tasks and does not address edge computing issues. Shi et al. [20] investigated a new A2ACS which included two UAV groups that utilized the collaborative beam forming technology to exchange data through the use of virtual antenna arrays. The study did not consider energy transfer. 

Although A2ACS have many advantages, their complexity and dynamics pose a great challenge to optimizing system performance. Traditional methods are not suitable for solving resource allocation in A2ACS because there are many constraints in such problems, such as accurate state information, dynamic conditions, and long-term optimization [21]. Multi-agent deep reinforcement learning (MADRL) can utilize the feature representation capability of deep neural networks to fit the state, action, and value functions to improve the performance of reinforcement learning models. It has been widely used in the field of UAV resource management ([22,23]). Liu et al. ([24,25]) proposed the deep Q-networks (DQN)-based multi-UAVs trajectory control strategy and the wireless communication network resource allocation method, which can effectively optimize the communication coverage and network throughput. However, the DQN algorithm uses a maximum-based estimation method to calculate the *Q*-value, which is prone to overestimating the *Q*-value, and also misleads the strategy selection, thus reducing the stability of training. Therefore, Peng et al. [26] developed an online path planning algorithm, based on the double deep Q-learning network (DDQN), and verified the performance advantages of this algorithm, in terms of convergence speed, the amount of offloaded data, and energy consumption. Ouamri et al. [27] proposed a multi-agent DQN(MADQN)-based optimization algorithm to optimize the energy efficiency of UAV-assisted device-to-device communication, to maximize throughput and energy efficiency. Unlike DQN and DDQN algorithms, which can only handle discrete action spaces, the deep deterministic policy gradient (DDPG) algorithm is used for continuous control tasks. Wang et al. [28] proposed a DDPG-based trajectory control algorithm to independently manage UAV trajectories and optimize the geographical distribution fairness, load fairness, and overall energy consumption among ground user devices. However, the proposed algorithm is only for air-to-ground communication scenarios, in which UAVs mainly serve stationary sensor devices on the ground, and thus the state space is relatively small. There is also the problem of building occlusion, which makes the transmission efficiency lower when considering non-line-of-sight channels. Yu et al. [18] developed an extended DDPG algorithm for learning UAV control strategies over multiple targets. Three objectives, including maximizing the data transfer rate of the ground devices, total energy harvesting, and minimizing the energy consumption of the UAV, were considered for a scenario in which the UAV uses a full-duplex mode to collect data from the ground target devices and charge other devices. This algorithm was also for air-to-ground communication scenarios and the optimization objective lacked prioritization considerations for devices in low-power state. Liu et al. [29] proposed a UAV path planning method, based on the DDPG algorithm, with multi-objective reinforcement learning. It enables the UAV to autonomously plan its path and satisfy multiple objectives during the mission. But this study does not take into account the energy transfer from the UAVs. Do et al. [30] investigated a wireless communication system for downlink communication driven by multiple UAVs, to maximize the service time and downlink throughput of the UAVs, using a deep reinforcement learning approach based on the DDPG algorithm. This study was also an air-to-ground communication scenario where UAVs were still serving users on the ground; only data transmission was considered in this scenario, without involving user task offloading and energy transfer.

### 1.2. Motivations and Contribution 

In this paper, we investigate a novel full-duplex A2ACS. Different from traditional air-to-ground communication systems, the objective of A2ACS is to provide continuous energy and necessary edge computation for multiple UAVs. Different from traditional single-objective or joint-objective optimization methods, we design a multi-objective optimization (MOO) framework for optimizing the service location and energy transmit power of air-edge energy servers (AEESs). By leveraging the MADRL approach, we develop a MADDPG-based multi-objective algorithm and a K-means-based clustering algorithm to solve the problem. The main contributions of this paper are summarized as follows:

- We propose a novel full-duplex A2ACS model combining WPT with MEC technology. This system uses AEESs to provide wireless charging services and edge computing for airborne UAVs. It can effectively reduce the computational delay and energy consumption of the UAVs, while ensuring that the UAVs will not interrupt the mission or leave the work area due to insufficient energy.
- We construct a MOO model for optimizing AEESs’ service location and energy transmit power. The model fully considers multiple objectives such as mission offloading, energy transfer, prioritization of UAVs, and energy consumption. We formulate four optimization objectives, including maximizing system throughput, minimizing the number of low-power UAV alarms, minimizing system energy consumption, and maximizing the energy transfer efficiency. The optimization objective weights can be adjusted according to the needs of real scenarios.
- We propose a decision-making algorithm as multi-objective deep deterministic policy gradient (MODDPG) based on the DDPG algorithm to achieve multi-objective optimization. We also propose a K-MAU (K-means for AEESs and UAVs) algorithm based on K-means clustering algorithm to determine the association between AEESs and UAVs, to ensure the fairness of the service.

The remainder of this paper is organized as follows. The system model and the A2AMOO problem are presented in Section 2. In Section 3, we propose the MODDPG algorithm and the K-MAU algorithm for UAV-assisted energy transfer and unloading. Simulated experiment results are shown in Section 4. Finally, Section 5 makes a conclusion.

## 2. System Model and Problem Formulation 

**Figure 1:**
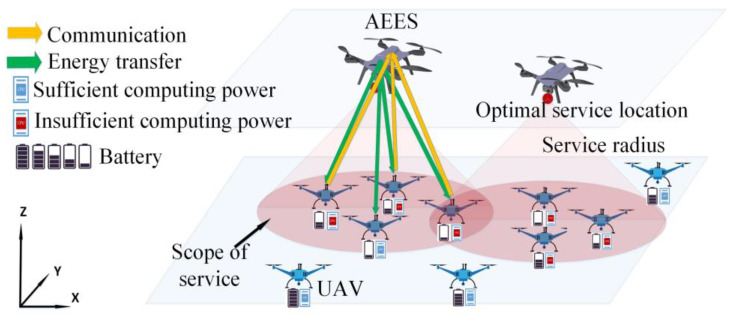
System model.

A full-duplex A2ACS based on MEC and WPT is illustrated in Figure 1. Assume that there is a set M≜{m=1,2,…,M} of UAVs that are supposed to move freely in a three-dimensional area of size $x\times y\;m^{2}$. The UAVs incur different resource demands with sustained energy consumption. To charge the UAVs and provide edge computing, we deploy another set N≜{n=1,2,…,N} of specific UAVs equipped with servers, RF energy transmitters, and large-size batteries. These specific UAVs are defined as air-edge energy servers (AEESs). For the fixed time duration, denoted as Γ, the AEESs provide edge computing and wireless charging services to the UAVs. To facilitate the calculation, the time duration is divided into T time slots, where τ=Γ/T is the length of time slot. Each time slot t∈T≜{t=1,2,…,T} is divided into sufficiently small time slots, so the position of the UAVs can be considered to be constant within a single time slot. To avoid collision between AEESs and UAVs, we assume that AEESs and UAVs are flying at the fixed altitudes of $H_{n}$ and $H_{m}$, respectively. To avoid signal interference and maintain a good transmission channel quality, we set the flight altitude difference between AEES and UAV as H. At each time slot, the AEES*n* and UAV*m* coordinate positions are defined as $L_{n}^{t}=[X_{n}^{t},Y_{n}^{t},Z_{n}^{t}]$ and $L_{m}^{t}=[X_{m}^{t},Y_{m}^{t},Z_{m}^{t}]$. Since the channel quality between AEESs and UAVs is negatively correlated with the transmission distance, the service coverage of AEESs is limited and the service coverage radius of AEESs is denoted as R. 

All AEESs constantly change their positions to find UAVs with low energy levels and high computational demands to charge them and provide edge computing. In addition, AEESs need to improve the energy transfer efficiency by controlling the energy transmit power $P_{n}^{t}$. To ensure that each UAV can only receive services provided from one AEES, we use the binary variable $b_{nm}^{t}\in \{0,1\}$ to denote the correspondence between AEES*n* and UAV*m*. $b_{nm}^{t}=1$ indicates that AEES*n* is associated with UAV*m* in time slot *t*, and $b_{nm}^{t}=0$ indicates that they are not associated.

### 2.1. Channel Model

**Figure 2:**
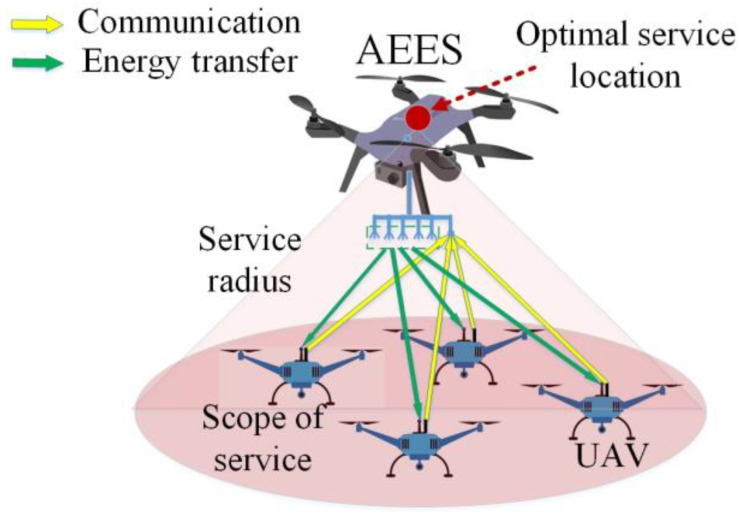
Channel model.

As shown in Figure 2, the AEESs fly to the optimal service location and work in full-duplex mode. Each AEES is equipped with A + 1 antennas. The first A antennas are used to generate multiple beams to transmit energy to the UAVs. Each beam covers a specific direction without overlapping. The AEES*n* sends RF signals to the UAVs through each beam with energy transmit power $P_{n}^{t}$. The (A + 1)th antenna is used for data transmission with UAVs.

Each UAV is equipped with two antennas operating in orthogonal frequency bands. One antenna is used for energy harvesting and the other antenna is used for data offloading.

**Figure 3:**
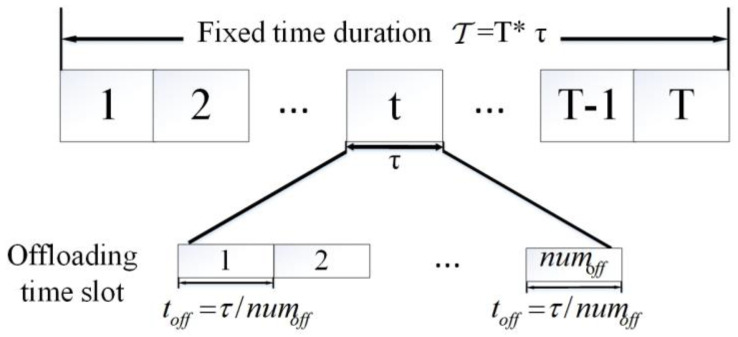
Time slot division.

To avoid channel interference between UAVs, we use the time division multiple access (TDMA) protocol to divide the data offloading time slots of each AEES [31], which can be seen in Figure 3. Assuming that the number of UAVs associated with AEES*n* at time slot *t* is represented by $num_{n}^{t}$, the UAV*m* is allocated an offloading time $t_{off}{}_{m}^{t}=\tau /num_{n}^{t}$. 

After receiving RF signals from the AEESs, the UAVs convert them into DC electrical energy to be stored in the battery. At the same time, the UAVs offload data to the AEESs using the communication channel. Considering that all flying devices have a certain altitude, we adopt the line-of-sight channel model to determine the AEES–UAV link. In the time slot *t*, the distance between AEES*n* and UAV*m* can be modeled as follows:

(1) $$|L_{n}^{t},L_{m}^{t}|=\sqrt{{\left(X_{n}^{t}-X_{m}^{t}\right)}^{2}+{\left(Y_{n}^{t}-Y_{m}^{t}\right)}^{2}+(H_{n}-H_{m}{)}^{2}},\forall n,m$$

#### 2.1.1. Energy Transfer Channel

The energy transfer channel between AEES*n* and UAV*m* can be expressed as follows [19]:

(2) $${\mathrm{CH}}_{nm}^{t}=\sqrt{g_{0}{\left(|L_{n}^{t},L_{m}^{t}|\right)}^{-\alpha }}b(\Omega )$$

where $g_{0}$ is the channel gain at a reference distance of 1 m and α is the path loss exponent [32]. We use a uniform linear array b(Ω) to represent the steering vectors for the elevation and azimuth angles of the LOS path, and b(Ω) can be expressed as:

(3) $$b(\Omega )={\left[1,\ldots ,e^{j\frac{2\pi agq}{c}\sin\Omega },\ldots ,e^{j\frac{2\pi Agq}{c}\sin\Omega }\right]}^{T}$$

where a∈{0,…,A−1} denotes the coordinates of the *a*th antenna, *c* is the speed of light, *q* is the transmission frequency, and g is the antenna spacing between the antenna elements. Therefore, the energy transfer channel gain between AEES*n* and UAV*m* is expressed as:

(4) $$g_{nm}^{t}=\frac{g_{0}}{{\left(|L_{n}^{t},L_{m}^{t}|\right)}^{\alpha /2}}|b^{H}(\Omega )U{|}^{2}$$

where $U=[u_{0},\ldots ,u_{a},\ldots ,u_{A}]$ denotes the beamforming vector describing the phase excitation and amplitude excitation of each array, i.e., $u_{a}=AE_{a}(\Omega )I_{a}e^{-j(2\pi agq/c)\sin\Omega }$, where $I_{a}$ and $AE_{a}(\Omega )$ are the amplitude excitation and the pattern of the *a*th array, respectively [19]. 

#### 2.1.2. Communication Channel

The communication channel gain for AEESs–UAVs can be modeled as:

(5) $$h_{nm}^{t}=\frac{g_{0}}{(|L_{n}^{t},L_{m}^{t}|{)}^{\alpha /2}}$$

assuming that UAV*m* has a transmission power of $P_{m}$. The channel bandwidth is *W* and the channel noise is $N_{0}$. The data transmission rate of UAV*m* in the time slot *t* is expressed as follows:

(6) $$R_{m}^{t}=b_{nm}^{t}W\log_{2}(1+\frac{P_{m}h_{nm}^{t}}{N_{0}})$$

### 2.2. Computing and Offloading Model

At each time slot, the UAVs receive computational tasks from ground users. The UAV needs to estimate whether it can fulfill the task with its local computational capability. The UAV*m* local computational capacity within a single time slot can be expressed as follows:

(7) $$M_{m}=\left(\frac{f_{m}}{C_{m}}\right)\times \tau$$

where $f_{m}$ is the CPU frequency, and $C_{m}$ is the number of required CPU cycles to compute one data bit. If the task size is larger than the computational capability, the UAV needs to offload the task to the corresponding edge server. The task offloading adopts the binary offloading strategy, i.e., either the task is entirely computed locally or the task is entirely offloaded to the server. 

Whether the offloading task of UAV*m* can be completed or not depends on the size of the offloading time slot provided by its associated AEES*n*. The amount of data that can be offloaded by UAV*m* in time slot *t* can be expressed as follows:

(8) $${C_{off}}_{m}^{t}=R_{m}^{t}\times {t_{off}}_{m}^{t}$$

If ${C_{off}}_{m}^{t}<D_{m}^{t}$, it means that the amount of data that can be offloaded by UAV*m* (denoted by $D_{m}^{t}$) does not meet the task requirements, and the task fails. If ${C_{off}}_{m}^{t}\geq D_{m}^{t}$ and the energy of UAV*m* is sufficient to complete the offloading task, the task succeeds.

### 2.3. Wireless Power Transfer and Energy Harvesting Model

During the entire service period, it is assumed that the AEESs have sufficient energy to provide services to the UAVs. When information from the target device is received through the uplink channel, the AEES continuously transmits power signals at a constant power $P_{n}^{t}$. According to the energy transfer channel gain in Equation (4), the received energy of UAV*m* within the time slot *t* can be represented by the following equation:

(9) $$Er_{m}^{t}=\sum_{n=1}^{N}\eta b_{nm}^{t}P_{n}^{t}g_{nm}^{t}\tau$$

where η∈(0,1) is the energy loss coefficient, $g_{nm}^{t}$ is the energy transfer channel gain between AEES*n* and UAV*m*, and τ is the length of the time slot.

### 2.4. Energy Consumption Model

This section analyzes the energy harvesting and consumption of AEESs and UAVs in a time slot.

#### 2.4.1. Changes in the Energy of Unmanned Aerial Vehicles

The energy of UAV*m* in the time slot *t* can be determined by three factors: the remaining energy $E_{m}^{t-1}$, the received energy $Er_{m}^{t}$, and the energy consumption $E\_out_{m}^{t}$. The energy consumption of UAV*m* consists of the propulsive energy consumption and the computational energy consumption.

Propulsive energy consumption is the main energy consumed by UAVs during flight. We reference the analytical model of existing rotary-wing UAVs [33] and assume that UAVs fly at a constant speed. The propulsive energy consumption of UAV*m* in the time slot *t* is calculated by:

(10) $$E_{m\_boost}^{t}=P1(1+\frac{3v_{m}{}^{2}}{v_{tip}^{2}})+P2\sqrt{\sqrt{1+\frac{v_{m}{}^{4}}{4v_{ind}^{4}}}-\frac{v_{m}{}^{2}}{2v_{ind}^{2}}}+\frac{1}{2}\zeta_{1}\zeta_{2}\zeta_{3}\zeta_{4}v_{m}{}^{3}$$

where $v_{m}$ is the flight speed of UAV*m*, $v_{tip}$ is the tip speed of the rotor, and $v_{ind}$ represents the average induced velocity of the rotor. Pi(i=1,2) respectively represents the blade power and induced power in hover. $\zeta_{i}(i=1,2,3,4)$ respectively represents the fuselage drag ratio, air density, rotor disk area, and rotor solidity.

The computational energy consumption is generated by either local computing or offloading. They are formulated as follows:

(11) $$E_{m\_com}^{t}=(1-off_{m}^{t})\lambda {\left(f_{m}\right)}^{3}\tau$$

(12) $$E_{m\_off}^{t}=off_{m}^{t}\frac{D_{m}^{t}}{R_{nm}^{t}}P_{m}$$

where $off_{m}^{t}=0$ represents a case where UAV*m* does not offload the task during time slot *t*, which leads to the local computational energy consumption. λ is the effective capacitance coefficient of the processor’s chip that is determined by the chip architecture [34], and $f_{m}$ is the CPU frequency of UAV*m*. $off_{m}^{t}=1$ represents a case where UAV*m* offloads the task during time slot *t*. From Equation (6), we know that $R_{nm}^{t}$ is the channel transmission rate between AEES*n* and UAV*m* during time slot *t*, $D_{m}^{t}$ is the task data size of UAV*m*, and $P_{m}$ is the power of UAV*m*. 

Apart from satisfying the sum of propulsive energy and computational energy, the energy consumption $E\_out_{m}^{t}$ must be less than the existing energy, i.e., $E\_out_{m}^{t}\leq E_{m}^{t-1}+Er_{m}^{t}$. Therefore, $E\_out_{m}^{t}$ can be obtained by the following equation:

(13) $$E\_out_{m}^{t}=\min(E_{m\_com}^{t}+E_{m\_off}^{t}+E_{m\_boost}^{t},\;E_{m}^{t-1}+Er_{m}^{t})$$

Furthermore, under the restriction of UAV battery capacity $B_{m}$, i.e., $E_{m}^{t}\leq B_{m}$, the energy of UAV*m* in the time slot *t* can be represented as:

(14) $$E_{m}^{t}=\min\{E_{m}^{t-1}+Er_{m}^{t}-E\_out_{m}^{t},B_{m}\}$$

#### 2.4.2. Changes in the Energy of Air-Edge Energy Servers

The energy change in AEES*n* can be determined by two parts: the remaining energy in time slot *t* − 1 and the energy consumed in time slot *t*. The energy consumed by AEES*n* includes the transfer energy consumption $Es_{n}^{t}$, the computational energy consumption $E_{n\_com}^{t}$, and the propulsion energy consumption $E_{n\_boost}^{t}$. 

AEES*n* provides wireless charging services to its associated UAVs with a constant transmit power $P_{n}^{t}$. The transfer energy consumption can be represented as follows:

(15) $$Es_{n}^{t}=P_{n}^{t}\tau$$

The edge computing energy consumption can be represented as follows:

(16) $$E_{n\_com}^{t}=\lambda {\left(f_{n}\right)}^{3}\tau$$

where λ is the computation consumption coefficient and $f_{n}$ is the CPU frequency of AEES*n*.

Similar to Equation (10), the propulsion energy consumption of AEES*n* can be obtained from the following equation:

(17) $$E_{n\_boost}^{t}=P1(1+\frac{3v_{n}{}^{2}}{v_{tip}^{2}})+P2\sqrt{\sqrt{1+\frac{v_{n}{}^{4}}{4v_{ind}^{4}}}-\frac{v_{n}{}^{2}}{2v_{ind}^{2}}}+\frac{1}{2}\zeta_{1}\zeta_{2}\zeta_{3}\zeta_{4}v_{n}{}^{3}$$

where $v_{n}$ is the flight speed of AEES*n*, $v_{tip}$ is the tip speed of the rotor, and $v_{ind}$ represents the average induced velocity of the rotor. Pi(i=1,2) respectively represents the blade power and induced power in hover. $\zeta_{i}(i=1,2,3,4)$ respectively represents the body drag ratio, air density, rotor disk area, and rotor solidity. Therefore, the energy consumed by AEESs in time slot *t* can be expressed as follows:

(18) $$E\_out_{n}^{t}=Es_{n}^{t}+E_{n\_com}^{t}+E_{n\_boost}^{t}$$

The energy of AEES*n* can be expressed as follows:

(19) $$E_{n}^{t}=E_{n}^{t-1}-E\_out_{n}^{t}$$

### 2.5. Problem Formulation

The primary goal of the A2ACS is to maximize the system task completion rate and throughput, maximize the total energy received by UAVs, and minimize the number of low battery warnings for UAVs while minimizing the energy consumption of all AEESs. On the one hand, due to the dynamic changes in the positions of AEESs and UAVs in each time slot, the channel quality also varies with the distance between them. Therefore, AEESs need to make decisions on optimal service location, denoted as $L_{n}^{t}=[X_{n}^{t},Y_{n}^{t}]$, to maintain higher quality channels, resulting in higher service coverage and prioritizing service to UAVs with low battery levels. On the other hand, to ensure the balance of energy transmission for UAVs and to prevent the overflow of battery capacity due to excessive energy reception, AEESs also need to make decisions on the energy transmit power, denoted as $P_{n}^{t}$, to ensure the highest energy transmission efficiency while reducing energy consumption.

- Optimization objective 1:

In this paper, the first objective is to maximize the system throughput. Based on a binary variable $com_{m}^{t}$, we can know whether the computation task of UAV*m* in the time slot *t* is completed. Therefore, the system throughput over the total service duration can be expressed as:

(20) $$C_{total}^{}=\sum_{t=1}^{T}\sum_{m=1}^{M}com_{m}^{t}D_{m}^{t}$$

where $D_{m}^{t}$ is the task size of the UAV*m*.

- Optimization objective 2:

Minimizing the number of low battery warnings for UAVs during the total service duration is the second objective. We assume that a low battery warning will be triggered when the battery level of a UAV drops below 15%. To avoid task failures caused by low battery of UAVs, AEESs should prioritize serving UAVs with lower battery levels. The cumulative number of low battery alarms for UAVs during the entire service period can be represented as follows:

(21) $$A_{total}=\sum_{t=1}^{T}\sum_{m=1}^{M}AL_{m}^{t}$$

where $AL_{m}^{t}$ is a binary variable. $AL_{m}^{t}=1$ indicates that a low battery warning was generated by UAV*m* in time slot *t*. Otherwise, $AL_{m}^{t}=0$.

- Optimization objective 3:

The third objective is to maximize the energy transfer efficiency between AEESs and UAVs, i.e., maximize the total energy received by UAVs. According to Equation (9), we can obtain the received energy $Er_{m}^{t}$ in time slot *t*. However, due to the limitation of the UAV battery capacity $B_{m}$, the effectively received energy $Ep\_r_{m}^{t}$ cannot exceed the battery capacity, i.e., $Ep\_r_{m}^{t}=\min\{Er_{m}^{t},B_{m}-E_{m}^{t-1}-E\_out_{m}^{t}\}$.

Therefore, the total energy effectively received by UAVs during the total service duration can be obtained by the following formula:

(22) $$E_{total}^{r}=\sum_{t=1}^{T}\sum_{m=1}^{M}Ep\_r_{m}^{t}$$

- Optimization objective 4:

Minimizing the energy consumption of all AEESs is the fourth objective. Reducing the energy consumption of the AEESs and ensuring the energy transmission efficiency can be achieved by controlling the energy transmit power of AEESs will improve the network’s lifetime. As the initial energy of AEESs is fixed, the effectiveness of power control can be approximately judged by the total electric surplus of AEESs after the total service time, which can be expressed as:

(23) $$E_{total}^{end}=\sum_{n=1}^{N}E_{n}^{T}$$

where $E_{n}^{T}$ is the energy of the last time slot T of AEES*n*, which is the electric surplus of AEES*n* after the total service time Γ. 

In summary, the A2AMOOP based on energy transmission and edge computing can be formulated as follows:

(24) $$P:\underset{\left(X_{1}^{t},\ldots ,X_{N}^{t},Y_{1}^{t},\ldots ,Y_{N}^{t},P_{1}^{t},\ldots ,P_{N}^{t}\right)}{\max}(C_{total},-W_{total},E_{total}^{r},E_{total}^{end})$$

s.t.

$$\begin{array}{l} C1:b_{nm}^{t}\in \{0,1\},\forall t\in T,\forall n\in N,\forall m\in M\\ C2:\sum_{n\in N}b_{nm}^{t}=1,\forall t\in T,\forall m\in M\\ C3:0\leq E_{m}^{t},\forall t\in T,\forall m\in M\\ C4:E\_out_{m}^{t}\leq E_{m}^{t-1}+Er_{m}^{t},\;\forall t\in T,\forall m\in M\\ C5:E_{m}^{t-1}-E\_out_{m}^{t}+Er_{m}^{t}<B_{m},\forall t\in T,\forall m\in M\\ C6:\left\{ \begin{array}{l} 0\leq X_{n}^{t}\leq x,\forall t\in T,\forall n\in N\\ 0\leq Y_{n}^{t}\leq y,\forall t\in T,\forall n\in N \end{array}\right.\\ C7:\left\{ \begin{array}{l} 0\leq X_{m}^{t}\leq x,\forall t\in T,\forall m\in M\\ 0\leq Y_{m}^{t}\leq y,\forall t\in T,\forall m\in M \end{array}\right.\\ C8:Pmin\leq Pn_{n}^{t}\leq Pmax,\forall t\in T,\forall n\in N\\ C9:|H_{m}-H_{n}|<R \end{array}$$

where $b_{nm}^{t}$ represents the association between AEES*n* and UAV*m* and C1 and C2 guarantee that each UAV is associated with only one AEES. C3, C4, and C5 ensure the correctness of the UAVs’ energy during the service period: C3 guarantees that UAVs’ energy is non-negative; C4 limits the energy consumption of the UAVs to not exceed the available energy; and C5 restricts the UAVs’ energy from exceeding the battery capacity. C6 and C7, respectively, limit the flight range of AEESs and UAVs in each time slot. To avoid irreparable damage to the capacitors and batteries of AEESs and UAVs, C8 limits the power range of AEESs. C9 ensures that the height difference between AEESs and UAVs does not exceed the service radius.

## 3. Deep Deterministic Policy Gradient-Based Method for Air-to-Air Multi-Objective Optimization Problem

The above A2AMOOP is a non-linear programming (NLP) problem. There is a deterministic algorithm to solve it in polynomial time. Furthermore, the mobility of AEESs and UAVs leads to high network dynamics. The problem needs to be resolved within a short time to satisfy the requirements of the UAVs. Therefore, we develop a deep reinforcement learning-based method to solve the problem.

Artificial intelligence algorithms have many advantages, including the ability to process large-scale data, discover patterns and rules in the data, and the ability to learn and optimize autonomously. In reinforcement learning, the agent takes rapid feedback from the environment and learns how to make decisions to maximize the reward. The agent observes the state $S^{t}$, performs the action $a^{t}$, and updates the state to $S^{t+1}$. This process is repeated until the end of the training. Deep learning is capable of handling large-scale and complex problems using multi-layer neural networks. Therefore, multi-agent deep reinforcement learning (MADRL) can learn near-optimal or equilibrium strategies in complex large-scale environments, realizing the decision-making of multiple decision-making subjects, which is very suitable for realizing adaptive control of multi-UAV systems.

DDPG is one of the classical MADRL algorithms for continuous control problems. In DDPG, the actor network maps states to actions and the critic network evaluates the value of the actions, based on the output of the actor network. Compared with other policy-based MADRL algorithms, the DDPG algorithm has the advantages of high training efficiency, high sampling efficiency, and ease of training. Therefore, we propose a DDPG-based method to solve the A2AMOO problem.

### 3.1. Problem Transformation

In this section, we re-model the proposed A2AMOO problem as a scalable Markov decision process (MDP). MDP can be represented by a tuple (S, A, R, f, γ), representing the state space, action space, reward space, transition probability space, and reward discount factor, respectively. Each AEES is an agent. Since there is no competition among AEESs, the agents are fully cooperative. Our model has the following three basic components:

#### 3.1.1. State Space

The state space describes the state of all the UAVs, including the positional coordinates, battery capacity, and time-varying task sizes. It can be expressed as:

(25) $$S^{t}=[X_{1}^{t},\ldots ,X_{M}^{t},Y_{1}^{t},\ldots ,Y_{M}^{t},E_{1}^{t},\ldots ,E_{M}^{t},D_{1}^{t},\ldots ,D_{M}^{t}]$$

where $\left(X_{m}^{t},Y_{m}^{t}\right)$ represents the position of UAV*m* at time slot *t*, $E_{m}^{t}$ denotes the battery capacity, and $D_{m}^{t}$ denotes the task size.

#### 3.1.2. Action Space

By observing the state, the AEESs take actions in real-time. The joint action is defined as:

(26) $$A^{t}=[A_{1}^{t},\ldots ,A_{N}^{t}]$$

where $A_{n}^{t}$ represents the action of AEES*n* at time slot *t*, and can be described as:

(27) $$A_{n}^{t}=[X_{n}^{t},Y_{n}^{t},P_{n}^{t}]$$

where $\left(X_{n}^{t},Y_{n}^{t}\right)$ represents the optimal service location of AEES*n* at time slot *t*, and $P_{n}^{t}$ determines the energy transmit power of AEES*n* at time slot *t*.

#### 3.1.3. Reward and Penalty

The design of the reward function can greatly affect the learning efficiency of agents. The goal of our reward function design is to improve the system throughput, prioritize low-power UAVs, reduce system energy consumption, and improve the energy transfer efficiency. Therefore, we have designed four reward elements with corresponding Equations (20)–(23), namely $C_{total}$, $A_{total}^{}$, $E_{total}^{r}$ and $E_{total}^{end}$. The reward is represented as a four-dimensional vector:

(28) $$R≜\{R^{t}\}=\{[r_{C}^{t},r_{A}^{t},r_{ER}^{t},r_{E}^{t}]\}$$

Furthermore, to avoid collisions due to AEESs at the same location, a penalty ρ is used to punish, in the case of a collision.

### 3.2. Multi-Objective Deep Deterministic Policy Gradient Algorithm 

**Figure 4:**
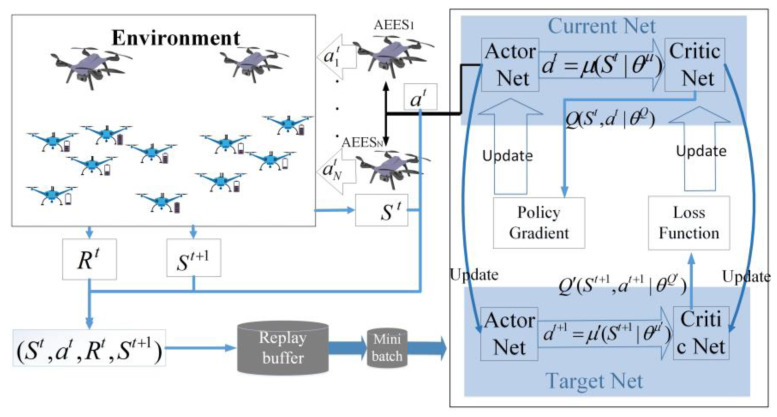
Structure of the MODDPG algorithm.

In this section, a MODDPG algorithm is proposed to solve the A2AMOO problem. The DDPG algorithm uses an actor network to learn the policy function, which outputs actions under a given state. Meanwhile, a critic network is used to evaluate the quality of the output of the actions by the actor network, which outputs the score of the actions. Each network has its corresponding target network, so the MODDPG algorithm includes four networks, namely the actor network $\mu (\cdot |\theta^{\mu })$, critic network $Q(\cdot |\theta^{Q})$, target actor network $\mu^{\prime }(\cdot |\theta^{\mu^{\prime }})$, and the target critic network $Q^{\prime }(\cdot |\theta^{Q^{\prime }})$, in which $\theta^{\mu }$, $\theta^{Q}$, $\theta^{\mu^{\prime }}$, and $\theta^{Q^{\prime }}$ represent the parameters of each network, respectively. As shown in Figure 4, AEESs are used as agents for communication with the UAVs. At time slot *t*, all AEESs observe the current environment state $S^{t}$ and input it into the actor network. By calculating the policy function $\mu (\cdot |\theta^{\mu })$, the action vector $a^{t}=[a_{1}^{t},\ldots ,a_{n}^{t}]$ is obtained. Then, the state to $S^{t+1}$ is updated and the reward $R^{t}$ is generated. Then, the state $S^{t}$ and action $a^{t}$ are input into the critic network to calculate the Q value through $Q(\cdot |\theta^{Q})$, describing whether taking the action is appropriate under the current state.

Specifically, the joint action $a=a_{1}\times \ldots \times a_{N}$ of the AEESs determines the next state and the reward. The goal of the system is to find the optimal policy $\pi_{}^{*}(s)=\arg\max_{\pi }Q_{n}^{\pi }(s,a)$, which chooses the optimal action in the current state, to maximize the expected total future discounted reward. We use $Q(s,a|\theta^{Q})$ and $Q^{\prime }(s^{\prime },a^{\prime }|\theta^{Q^{\prime }})$ to represent the approximate *Q*-value and its target *Q*-value, which are defined as follows:

(29) $$Q_{\mu }(s,a)=\{\sum_{k=0}^{\infty }\gamma^{k}R^{{}_{k+t+1}}|s,a\}$$

where γ is the discount factor, which represents the discounted contribution of the current state to future states. The MODDPG algorithm adopts a delayed update strategy; that is, the parameter updates of the actor and critic networks are not synchronized, but alternate. The parameter update of the critic network depends on the temporal difference error, and updates $\theta^{Q}$ by minimizing the loss function. The loss function is used to measure the error between the predicted *Q*-value and the target *Q*-value, and can be expressed as:

(30) $$L(\theta^{Q})=\frac{1}{sum_{i}}\sum_{i}[(R^{i}+\gamma Q^{\prime }(S^{i+1},\mu^{\prime }(S^{i+1}|\theta^{\mu^{\prime }})|\theta^{Q^{\prime }}))-Q(S^{i},a^{i}|\theta^{Q}){]}^{2}$$

where $sum_{i}$ represents the total number of samples, and the critic network needs to be constantly optimized in each iteration of the training process, minimizing the loss function. To ensure computational efficiency, a batch gradient descent algorithm is used to optimize the loss function and update weight parameters. The parameter update of the actor network depends on the output of the critic network, which trains the policy network by maximizing the *Q*-value estimate of the critic network on the action output of the actor network. The actor network parameter is updated, according to the deterministic policy gradient ascent strategy, which can be represented as:

(31) $$\nabla_{\theta^{\mu }}J(\mu )\approx \frac{1}{sum_{i}}\sum_{i}\left(\nabla_{a}Q(S^{i},a^{i}|\theta^{Q})\nabla_{\theta^{\mu }}\mu (S^{i}|\theta^{\mu })\right)$$

During the training process, the two target networks are updated using the exponential smoothing method, and the target network parameter update methods are shown in the following equations:

(32) $$\theta^{Q^{\prime }}\leftarrow tau\theta^{Q}+(1-tau)\theta^{Q^{\prime }}$$

(33) $$\theta^{\mu^{\prime }}\leftarrow tau\theta^{\mu }+(1-tau)\theta^{\mu^{\prime }}$$

where the parameter 0<tau<<1 is used to ensure that the target network is updated slowly and steadily, which improves the stability, $\theta^{\mu }$, of learning.

The detailed steps of the MODDPG algorithm based on DDPG are shown in Algorithm 1. During the training process, the first step is to initialize the number of training epochs $episode_{max}$ and the number of training steps. We also initialize the buffer $M_{n}$ in line 1. In lines 2 and 3, we initialize the actor network, critic network, target actor network, target critic network, and their respective parameters. We also initialize the noise-related parameters in line 4.
**Algorithm 1:** MODDPG Algorithm**Input:**$\;a\;\mathrm{weight}\;\mathrm{vector}\;w=[w_{c},w_{A},w_{ER},w_{E}]$**.****Initialize:**0: Initialize the number of training epochs episode_max and the number of training steps T.$1:\;\mathrm{Initialize}\;\mathrm{replay}\;\mathrm{buffer}\;M_{n}$$,\;\mathrm{where}\;(M_{n}\neq \Phi$);$2:\;\mathrm{Randomly}\;\mathrm{initialize}\;\mathrm{Actor}-\mathrm{network}\;\mathrm{and}\;\mathrm{Critic}-\mathrm{network}\;Q(\cdot |\theta^{Q})$;$3:\;\mathrm{Initialize}\;\mathrm{target}\;\mathrm{networks}\;\mu^{\prime }(\cdot |\theta^{\mu^{\prime }})$$\;\mathrm{and}\;Q^{\prime }(\cdot |\theta^{Q^{\prime }})$;$4:\;\mathrm{Initialize}\;\mathrm{the}\;\mathrm{action}\;\mathrm{noise}\;\varepsilon =\varepsilon_{\max}$$\;\mathrm{and}\;\mathrm{noise}\;\mu (\cdot |\theta^{\mu })$$e\;\mathrm{decay}\;\mathrm{rate}\;\mathrm{is}\;\varepsilon_{decrease}$;$5:\;\mathrm{for}\;episode\;\leftarrow \;1\;\ldots \;{\mathrm{episode}}_{\max}$ **do**6:   $\;\mathrm{Initializes}\;\mathrm{state}\;S^{0}=[X_{1}^{0},\ldots ,X_{M}^{0},Y_{1}^{0},\ldots ,Y_{M}^{0},E_{1}^{0},\ldots ,E_{M}^{0},D_{1}^{0},\ldots ,D_{M}^{0}]$;7:    **for**
*t* ← 1…T **do**8:       Gradually reducing exploration probability:        $\mathrm{update}\;\mathrm{according}\;\mathrm{to}\;\varepsilon =\varepsilon_{\max}-\varepsilon_{decrease}$;9:       $a^{t}=a_{1}^{t}\times \ldots \times a_{N}^{t}=\mu (s^{t}|\theta^{\mu })+N(0,\varepsilon )$;10:       get b[n][m] according to Algorithm 2;11:      $\;\mathrm{AEES}n\;\mathrm{executes}\;\mathrm{the}\;\mathrm{action}\;a_{n}^{t}=[X_{n}^{t},Y_{n}^{t},P_{n}^{t}]$, n∈N;12:      $\;\mathrm{Observe}\;\mathrm{the}\;\mathrm{reward}\;R^{t}$$\;\mathrm{based}\;\mathrm{on}\;(28),\;\mathrm{and}\;\mathrm{get}\;\mathrm{the}\;\mathrm{new}\;\mathrm{state}\;S^{t+1}$;13:      $\;\mathrm{Store}\;(S^{t}$$,\;a^{t}$$,\;R^{t}$$,\;S^{t+1}$$)\;\mathrm{in}\;\mathrm{replay}\;\mathrm{memory}\;M_{n}$ ;14:       **If** updated **then**15:         $\;\mathrm{Randomly}\;\mathrm{sample}\;a\;\mathrm{mini}-\mathrm{batch}\;\mathrm{transitions}\;D_{n}$$\;\mathrm{from}\;M_{n}$;16:         $\;\mathrm{Update}\;\mathrm{parameters}\;\mathrm{of}\;\mathrm{the}\;\mathrm{Critic}-\mathrm{network}\;\theta^{Q}$ by minimizing its loss function according to Equation (30);17:         $\;\mathrm{Update}\;\mathrm{parameters}\;\mathrm{of}\;\mathrm{the}\;\mathrm{Actor}-\mathrm{network}\;\theta^{\mu }$by using the policy gradient approach according to Equation (31);18:         $\;\mathrm{Update}\;\mathrm{the}\;\mathrm{corresponding}\;\mathrm{target}\;\mathrm{network}\;\mathrm{parameters}\;\theta^{Q^{\prime }}$$\mathrm{and}\;\theta^{\mu^{\prime }}$ by Equations (32) and (33);19:    **end for**20: **end for**

**Algorithm 2:** K-means for AEESs-UAVs (KMID)**Input:**$\mathrm{The}\;\mathrm{location}\;\mathrm{of}\;\mathrm{AEESs}\;L_{n}$(n∈N$)\;\mathrm{and}\;\mathrm{the}\;\mathrm{service}\;\mathrm{location}\;\mathrm{of}\;\mathrm{UAVs}\;L_{m}$(m∈M).**output:** The relation matrix **b[n][m]** of AEESs and UAVs.
**Begin**
1: **Initialize: b[n][m]=0**

$2:\mathrm{Initialize}\;\mathrm{clustering}\;\mathrm{centers}:\;C_{n}=L_{n},\forall n\in Ν$

3: **for**
*i* ← 1 : Iterations **do**4:   **for**
*n* ← 1 : N **do**5:     **for**
*m* ← 1 : M **do**6:     $g_{nm}^{},h_{nm}$ ← Calculate the energy transfer channel gain and communication channel gain between AEES*n* and UAV*m*7:     **end for**8:**   end for**9:**   for**
*m* ← 1 : M **do**10:    UAV*m* joins the cluster of AEES*n* that is closer in energy transfer channel gain and communication channel gain:     $\mathrm{joins}\;\mathrm{the}\;C_{n}\;\mathrm{according}\;\mathrm{to}\;\arg\max(g_{nm}+h_{nm})$11:**  end for**12:  **for**
*n* ← 1 : N **do**13:    $\mathrm{Calculate}\;\mathrm{the}\;\mathrm{new}\;\mathrm{clustering}\;\mathrm{centers}:\;C_{n}=\frac{1}{\left|c_{n}\right|}\sum_{UAV_{m}\in c_{n}}L_{n},\forall m\in M$14:**  end for**
15:**  If**
$C_{n}$ no longer changes **then** end the loop.16: **for**
*n* ← 1 : N **do**17:  **for**
*m* ← 1 : M **do**18:**    If** UAV*n*∈$c_{1}$ **and** $\left|L_{n},L_{m}\right|$$<=R\;:\;b_{nm}^{}=1$19:**    Else** : $b_{nm}^{}=0$20:  **end for**21: **end for**

At the beginning of each episode, we initialize the environment and obtain the initial state $S_{0}$ in line 6. In line 8, ε decays at a rate of $\varepsilon_{decrease}$ at each time step. In line 9, a random noise N(0,ε) is added to the actions, which allows the agent to explore the environment. In line 10, AEESs obtain actions through the actor network, and then use Algorithm 2 to determine their association with UAV users. Each AEES executes $a_{n}^{t}$, updates the environment state, and obtains a reward (lines 11 and 12). The experience is stored in the replay buffer $M_{n}$ in line 13. 

Then, in lines 15–18, a random mini-batch samples $D_{n}$ tuples from the buffer $M_{n}$ to make the updated versions of the four networks. We use the loss function in Equation (30) and the policy gradient in Equation (31) to update the actor network and critic network, respectively. Additionally, we update the target actor network and target critic network using Equation (32) and Equation (33), respectively.

### 3.3. K-means for Air-Edge Energy Servers and Unmanned Aerial Vehicles (K-MAU)

The k-means clustering algorithm is a classical algorithm that can classify the data points into different clusters and find the center point of the clusters to represent the clusters. Compared to other algorithms, the K-means clustering algorithm is fast, simple, and easy to understand, so it is widely used in research [35]. The algorithm is commonly used to group and manage UAVs ([36,37]). When multiple UAVs collaborate on a task, the randomness of UAV paths may lead to overlapping services and resource wastage. To avoid this, clustering and categorizing all UAVs and ensuring that each cluster can only receive services provided by one AEES is required, to avoid the overlapping of resources. In addition, to improve the system efficiency, clustering can be used to maintain a higher-quality energy transfer channel between the AEES and UAVs on the one hand, and a higher-quality communication channel between the AEES and UAVs on the other hand. Therefore, in this section, we propose a K-means based clustering algorithm, to determine the service relationship between AEESs and UAVs.

The K-MAU algorithm aims to divide the UAVs into different clustering groups, so that each UAV can be associated with an AEES. More specifically, if one UAV is covered by multiple AEESs, it is important to choose one AEES to improve the overall channel quality and keep the fairness of resource allocation. In each time slot, after the AEESs determine the location of UAVs using Algorithm 1, the K-means clustering algorithm is used to calculate the service relation between the AEESs and UAVs. 

The input of the algorithm is the three-dimensional positions of all AEESs and UAVs, denoted as $L_{n}$(n∈N) and $L_{m}$(m∈M), respectively. As output, it returns the relation matrix of AEESs and UAVs, represented as b[n][m]. The detail of K-MAU is shown in Algorithm 2. First, the cluster centers are initialized by the AEESs’ positions. Then, the energy transfer channel gain and communication channel gain between all AEES*n* and UAV*m* are calculated. Each UAV is assigned to the AEES cluster center with the highest channel gain. After that, the new cluster centers are calculated based on the UAVs’ positions in each cluster. The process repeats until $C_{n}$ no longer changes. Finally, based on the clustering result, the service relation matrix is determined. 

## 4. Simulation Results 

In this section, we conduct a large number of experiments to evaluate the performance of the proposed algorithm. First, we present the experiment setup. Then, we show the performance of our proposed method by comparing it with other methods. 

### 4.1. Simulation Settings

We consider two AEESs and ten UAVs in a 100 m × 100 m region, and that a set **M** of UAVs is randomly moving and hovering within the target area, following the Gaussian–Markov model [19]. We set a safety distance of 0.5 m to ensure no collision between UAVs. By basing calculations on the current UAV’s position and speed information, as well as the UAV’s position prediction, our algorithm is able to adjust the direction of the UAV’s movement in a timely manner to avoid potential collisions. The difference in altitude between AEESs and UAVs is 10 m [38]. We set the mission period to 100 s and divide it into 100 time slots. The flight speed of the AEESs and the UAVs is 5 m/s. Since the time slot is tiny, it can be assumed that the positions of AEESs and UAVs remain unchanged during a time slot. The task size is between one and ten MBits. The total task size in each time slot is 40 MBits. The maximum energy of a UAV is 50 J. The emission power of AEES is between 40 W and 50 W. Other parameters such as path loss exponent, channel noise, and antenna gain are set according to previous works [19,31]. The parameters are summarized in Table 1.

The actor–critic network consists of four fully connected layers. A rectified linear unit (ReLU) is used as the activation function. The actor network was trained by using RMSProp Optimizer with a learning rate of 0.001, while the critic network was trained by using the Adam Optimizer with a learning rate of 0.001. The function is used as the output layer. The algorithms are implemented with Python 3.9 and PyTorch 1.8. The training parameters are shown in Table 2.

### 4.2. Results and Analysis

To evaluate the performance of the UAV-assisted WPT system, our proposed algorithm MODDPG is compared with the existing MADRL algorithm and the baseline methods. To comprehensively evaluate the performance of the MODDPG algorithm, we study the effects of the coverage radius of AEES, task size, and number of UAVs. The deep reinforcement learning algorithms include the double deep Q-network algorithm (denoted as MODDQN [26]) and the deep Q-network algorithm (denoted as MODQN [25]). The baseline algorithms include the genetic algorithm (denoted as MOGA), the random algorithm (denoted as MORA), and the greedy algorithm (denoted as MOGrA).

#### 4.2.1. Convergence Analysis

**Figure 5:**
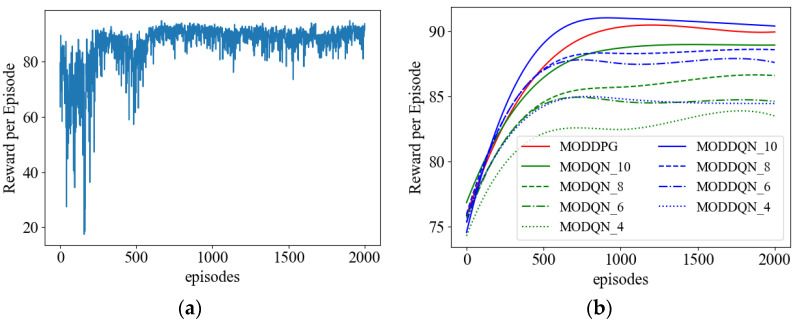
Reward per episode. (**a**) Cumulative reward. (**b**) Average cumulative rewards across MADRL algorithms after smoothing.

Figure 5a shows the convergence of our proposed MODDPG algorithm. According to Figure 5a, we can observe that the reward of MODDPG is very unstable in the initial stage, which is because, at the beginning of training, the agent has not gained enough experience to make accurate decisions. As the number of episodes increases, the agent gradually accumulates experiences by interacting with the environment and optimizes its strategy. As a result, the reward gradually increases and the algorithm converges. After 700 episodes of training, the reward reaches the maximum value and remains stable.

We compare MODDPG with DQN and DDQN in Figure 5b. According to Equations (26) and (27), we can see that the joint action of the two AEESs can be formulated as a 6-dimensional vector $A^{t}=[X_{1}^{t},Y_{1}^{t},P_{1}^{t},X_{2}^{t},Y_{2}^{t},P_{2}^{t}]$. Each dimension of MODQN and MODDQN is discretized by 4, 6, 8, and 10, denoted by MODQN_4, MODDQN_4, MODQN_6, MODDQN_6, MODQN_8, MODDQN_8, MODQN_10, and MODDQN_10, respectively. To better demonstrate the convergence of the MADRL algorithm, details are not a concern. We record the average reward per 100 rounds of training and smooth the curves to improve clarity.

It can be seen from Figure 5b that all the algorithms gradually converge as the number of training rounds increases. MODDPG performs well in terms of convergence speed and reward maximization. In contrast, the convergence and reward of MODDQN and MODQN are affected by the discretization of action spaces. As the number of discretization increases, the agent selects actions more accurately and thus obtains higher rewards.

#### 4.2.2. Comparison of Training Time and Model Size

Furthermore, we compare the training time and model size of MADRL algorithms in Table 3. Since the number of discretization are 4, 6, 8, and 10, the action spaces are $4^{6}$, $6^{6}$, $8^{6}$, and $10^{6}$, respectively. The action space of MODDPG is equal to the number of action dimensions.

According to Table 3, the training time increases as the action spaces increase, mainly because more combinations of actions need to be explored. The model size also increases as the action space increases because the neural network needs to fit more action values and corresponding *Q* values.

The result shows that MODDPG has advantages, in terms of its short training time and small model size. Since MODQN_8, MODDQN_8, MODQN_10, and MODDQN_10 have large model sizes, they are difficult to apply in real UAV scenarios. In contrast, MODQN_4 and MODDQN_4 have poor performances. Therefore, we choose MODDQN_6 and MODQN_6 models (referred to as MODDQN and MODQN) in the subsequent experiments.

#### 4.2.3. Comparison of Metrics during the Training Process

We consider the following metrics to compare the performance of the algorithms:

(a) System throughput: to indicate the number of tasks accomplished by all UAVs per time slot.

(b) Energy level of AEESs: to indicate the ratio of remaining energy to the total energy of AEESs.

**Figure 6:**
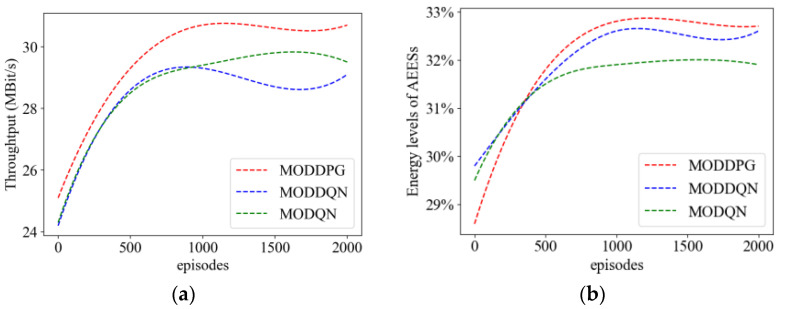
Performance comparisons per episode. (**a**) Throughput. (**b**) Energy levels of AEESs.

Figure 6 shows the comparison result of three metrics in the training process. From Figure 6a, it can be seen that as the number of training episode increases, the throughput of the three MADRL methods gradually increases. Among them, MODDPG has the fastest growth rate and reaches a stable peak of 28 MBit/s after 700 episodes. As can be seen in Figure 6b, the MODDPG algorithm achieves a higher energy level of AEESs than MODQN and MODDQN, which means that the MODDPG algorithm can make AEESs save more energy.

#### 4.2.4. Impact of Air-Edge Energy Server Coverage

In this subsection, we compare our proposed MADRL methods with baseline methods (MOGA, MORA, and MOGrA). Among them, the MORA algorithm randomly determines the location and transmit power of the AEESs at each time slot, while the MOGrA algorithm aims to maximize the number of UAVs covered by each AEES.

**Figure 7:**
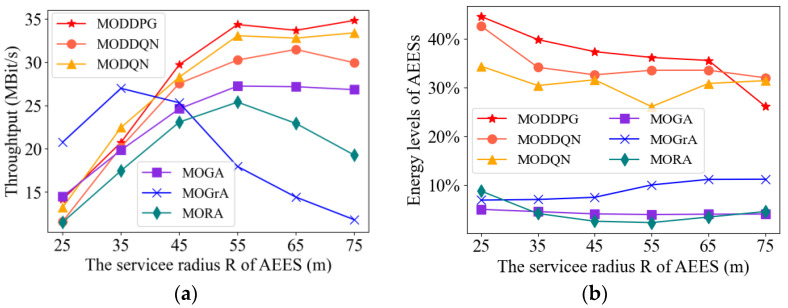
Performance comparison of the algorithms under different AEES coverage radius. (**a**) Throughput. (**b**) Energy levels of AEESs.

Figure 7 shows the performance of three metrics by varying the service radius. In Figure 7a, the throughput of MODDPG is significantly better than the other methods. In most of the methods, the throughput increases as R increases. This is because, as the R increases, the chances of UAVs accepting services increases. It is worth noting that the throughput of MOGrA increases and then decreases as R increases. This can be explained by the fact that MOGrA makes AEESs fly towards places with more UAVs, which leads to the offloading time slot being too small to satisfy the demand.

As seen from Figure 7b, compared to the three baseline methods, the energy levels of AEESs in the three MADRL methods are significantly higher. By effectively controlling the energy transmit power of the AEESs, the MADRL methods achieve the minimization of energy consumption. Among them, MODDPG has the best performance, and MODQN is slightly lower than MODDQN. This is because MODDQN improves the performance by using two neural networks for selecting actions and estimating the *Q*-values.

The MODDPG algorithm simultaneously considers several performance metrics, such as service coverage and energy efficiency. Optimization for the continuous action space makes the MODDPG algorithm find a better balance between weighing different objectives and performing better.

#### 4.2.5. Impact of Total Task Size

**Figure 8:**
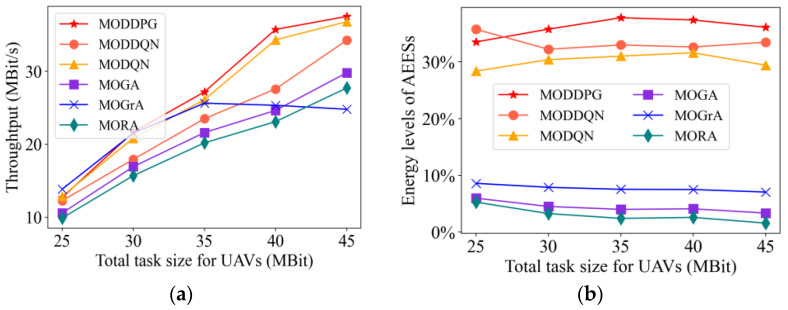
Performance comparison of the algorithms under different total task sizes of UAVs. (**a**) Throughput. (**b**) Energy levels of AEESs.

In Figure 8, we exhibit the metrics versus the total task size of UAVs. From Figure 8a, when the total task size is small, the difference between the system throughput is not significant. However, as the task size increases, the system throughput increases, while the gap becomes large. The MODDPG method has the highest system throughput compared to other methods.

According to Figure 8b, the total task size does not have a significant effect on the energy level of the AEES. Meanwhile, the AEES energy levels of the three MADRL methods are significantly higher compared with the three baseline methods. Among these three MADRL methods, the AEES energy level of the MODDPG method is the highest.

#### 4.2.6. Impact of Unmanned Aerial Vehicle Numbers

**Figure 9:**
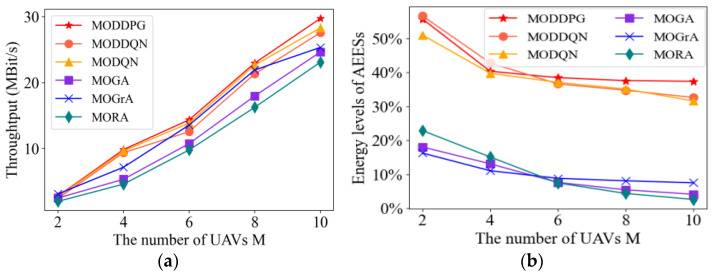
Performance comparison of the algorithms under different numbers of UAVs. (**a**) Throughput. (**b**) Energy levels of AEESs.

In Figure 9, we study the system’s performance under different methods by setting different numbers of UAVs. From Figure 9a, the system throughput of the methods increases as the number of UAVs increases. When the number of UAVs is small, it is hard to see the difference between the system throughputs of the methods. However, as the number of UAVs increases, the gap between the methods gradually increases. Note that the MODDPG method maintains the best performance at all quantities. The MODDPG method could adapt to scenarios with both enough resources and few resources.

According to Figure 9b, we can observe that as the number of UAVs increases, the AEES energy level decreases accordingly. To serve more UAVs, AEESs must consume more energy. By continuously adjusting the strategies of an AEES based on environmental feedback, the MADRL algorithms perform better in terms of energy control compared to the baseline approach. In contrast, the baseline methods lack full consideration of optimization objectives. The MODDPG method can control the energy transmit power of AEES more effectively than other methods.

The MODDPG method is able to manage the energy consumption of UAVs more intelligently, by focusing on the fairness of charging through the optimization process of deep reinforcement learning, thus reducing the low-power state of the system.

Taken together, the number of UAVs affects the performance of system throughput and AEES energy consumption. MODDPG algorithm focuses on the balance of multi-dimensional optimization objectives, which can achieve higher throughput and energy level through power control and positional decision-making.

#### 4.2.7. Comparison of Optimization Goals

**Figure 10:**
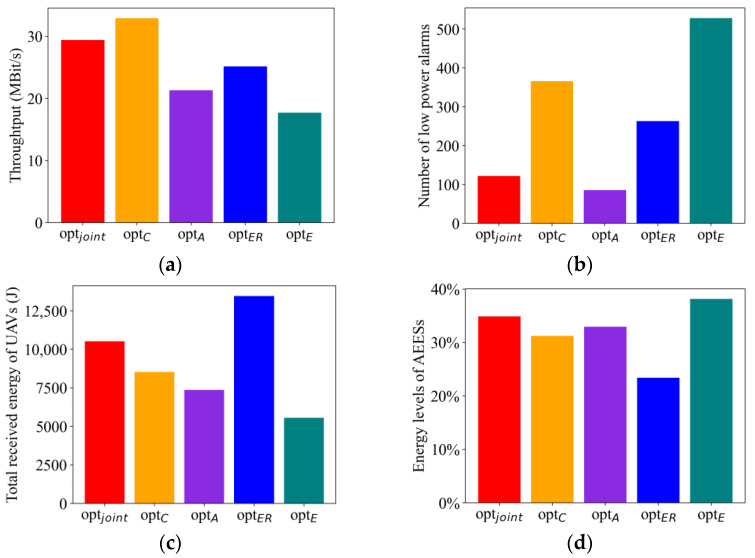
Optimized results under different policies: (**a**) throughput. (**b**) The number of low-power alarms of UAVs. (**c**) The received energy of UAVs. (**d**) Energy levels of AEESs.

We verified that the optimal policy is tuned to the weight parameters. The comparison experiment parameters are shown in Table 4. To compare the effects of different weight settings on the optimal strategies, in “$opt_{C}$”, “$opt_{A}$”, “$opt_{ER}$” and “$opt_{E}$”. We set the weights related to system throughput, low-power alarm, total harvested energy, and energy consumption to 1.0, and the weights of the other three objectives to zero. By comparing the multi-objective optimization strategy that considers four optimization objectives at the same time, “$opt_{jo\mathrm{int}}$”, with the four single-objective optimization strategies that optimize only a single objective, we obtained the optimization results of five different strategies, which are shown in Figure 10.

Figure 10a shows the system throughput under different optimization strategies, and it can be seen that the “$opt_{C}$” strategy can obtain the highest system throughput, which means that the system can handle more tasks or data volumes under this strategy. The throughput of “$opt_{jo\mathrm{int}}$“ strategy is slightly lower than “$opt_{C}$”. This is because in joint optimization methods, a balance needs to be struck between multiple optimization objectives, and the trade-offs between the optimization objectives may result in the throughput being affected. From Figure 10b, it can be seen that the total number of low-power alarms is the lowest in the “$opt_{A}$” strategy. The total number of low-power alarms in the “$opt_{jo\mathrm{int}}$” strategy is slightly higher than “$opt_{A}$”. The total number of low-power alarms in the other three strategies is much higher than “$opt_{A}$” and “$opt_{jo\mathrm{int}}$” because their optimization objectives do not consider prioritizing the service of UAVs in low-power states. Combining Figure 10a,c, the UAVs in “$opt_{ER}$” can obtain higher energy, but with lower throughput. This is because the AEESs trying to approach the UAVs may be associated with covering more UAVs, resulting in each UAV being allocated too small a slice of time to complete the task, thus reducing the throughput. From Figure 10d, it can be observed that “$opt_{E}$” achieves the lowest AEES energy consumption. This is because the AEES controls the amount of energy-emitted power and brings it closer to the target device, to conserve more energy. Combining Figure 10c,d, the UAV in “$opt_{ER}$” can obtain higher energy, but also has the highest AEES energy consumption. It is because in order for the UAV to obtain higher energy, the energy transmit power of the AEES needs to be increased, which will inevitably increase the energy consumption of the AEES.

The comparison results show that the MODDPG proposed in this paper can produce optimal policies under different preference conditions. Whether the preference is to maximize system throughput, maximize energy harvesting, or minimize energy consumption, the algorithm proposed in this paper can generate optimal strategies under different optimization objectives.

#### 4.2.8. Performance of K-MAU Algorithm

**Figure 11:**
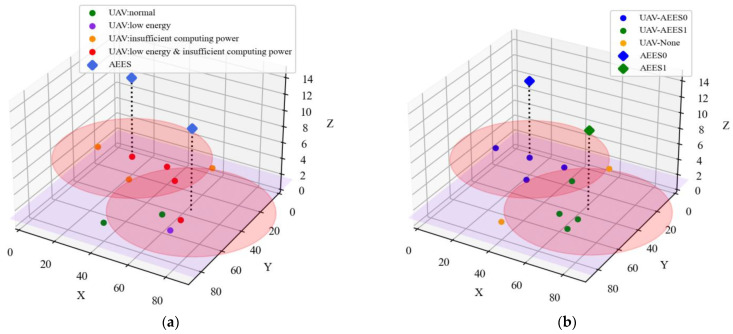
Performance of K-MAU algorithm. (**a**) Three-dimensional coordinate distribution of AEESs and UAVs. (**b**) Association relationship between AEESs and UAVs.

To clearly show the distribution of AEESs and UAVs as well as the service association relationship, we can present it using 3D images. In Figure 11a, the trained AEESs can intelligently select the best service location. It can be seen that the AEESs will prioritize flying near the UAVs with insufficient energy and computational capacity, based on their state information. By satisfying their charging and offloading needs, the AEESs prevent UAVs from crashing due to battery energy depletion or mission failure due to insufficient computing power. In Figure 11b, we show the use of the K-MAU algorithm to delineate the service association relationship between AEESs and UAVs. Each AEES is considered as a clustering center and each UAV is assigned to associate with the AEES closest to it, thus forming different clusters. It can be seen that this approach ensures that each UAV is service-associated with, at most, one AEES, while at the same time allowing them to be segmented into closer AEES ranges to provide better quality channel connectivity.

## 5. Conclusions

In this study, an air-to-air full-duplex communication system based on WPT and MEC technologies is proposed. The system provides continuous energy replenishment and edge computing support to UAVs. The problem is formulated as a multi-objective optimization problem aiming to optimize the system throughput, total harvested energy, number of low-power alerts for UAVs, and energy consumption. To achieve the optimization objective, we propose a multi-agent based MADRL method named MODDPG and design the reward function as a multi-dimensional vector corresponding to the optimization objective. To ensure service fairness and improve channel quality, a clustering algorithm, called K-MAU, is employed to determine the service association between AEESs and UAVs. The simulation results show that the MODDPG method outperforms the DDQN, DQN, and baseline methods, in terms of system throughput and energy level of AEESs. Furthermore, MODDPG can generate optimal policies based on different optimization objective weights. In future works, we plan to investigate the application of AEESs and UAVs in unfixed altitude scenarios, to further enhance the system performance.

## Figures and Tables

**Table 1:** Simulation setup.

Parameters	Value	Parameters	Value
Altitude of AEES*n* $H_{n}$	90 m	Flight speed of AEES*n* $v_{n}$	5 m/s
Altitude of UAV*m* $H_{m}$	100 m	Flight speed of UAV*m* $v_{m}$	5 m/s
Number of AEESs N	2	Battery capacity of AEES*n* $B_{n}$	8000 J
Number of UAVs M	10	Battery capacity of UAV*m* $B_{m}$	50 J
Time duration Γ	100 s	Transmit power of UAV*m* $P_{m}$	1.5 W
Number of time slots T	100	The speed of light c	3 × 10^8^ m/s
Length of time slot τ	1 s	Reference channel gain $g_{0}$	−30 dB
Length of area x	100 m	Communication bandwidth W	5 × 10^6^ Hz
Width of area y	100 m	Energy loss factor η	0.6
Service radius of AEESs R	50 m	CPU frequency of AEES*n* $f_{n}$	10 GHz
Minimum transmit power for AEESs $P_{\min}$	40 W	CPU frequency of UAV*m* $f_{m}$	500 MHz
Maximum transmit power of AEESs $P_{\max}$	50 W	CPU consumption factor λ	10^−27^
Path loss index α	2.2	Process density $C_{m}$	10^3^ cycles/bit
Transmission frequency of AEESs q	500 MHz		

**Table 2:** Key parameters of the training stage.

Parameters	Value	Parameters	Value
Memory size $M_{n}$	20,000	Actor learning rate	0.001
Mini-batch size $B_{s}$	128	Maximum exploration rate $\varepsilon_{\max}$	0.99
Steps for updating target	100	Reward decay rate γ	0.9
Steps for learning	30	Decreasing rate of exploration $\varepsilon_{decrease}$	0.0002
Optimizer method	Adam	Target soft update tau	0.25
Optimizer method	RMSProp	Number of neurons of hidden layer in the actor	[128, 256]
Critic learning rate	0.001	Number of neurons of hidden layer in the critic	[128, 256]

**Table 3:** Training of different MADRL algorithms.

Name	MODDPG	MODDQN_10	MODDQN_8	MODDQN_6	MODDQN_4	MODQN_10	MODQN_8	MODQN_6	MODQN_4
Action Space Size	6	$10^{6}$	$8^{6}$	$6^{6}$	$4^{6}$	$10^{6}$	$8^{6}$	$6^{6}$	$4^{6}$
Training Time (min)	18	52	28	22	20	43	37	22	23
Model Size (M)	0.71	247.97	65.01	11.05	1.03	247.97	65.01	11.05	1.03

**Table 4:** Parameters for different optimization objectives.

Name	$\mathrm{Parameters}\;w=[w_{c},w_{A},w_{ER},w_{E}]$
$opt_{jo\mathrm{int}}$	$w_{c}=1.0,\;w_{A}=1.0,\;w_{ER}=1.0,\;w_{E}=1.0$
$opt_{C}$	$w_{c}=1.0,\;w_{A}=0,\;w_{ER}=0,\;w_{E}=0$
$opt_{A}$	$w_{c}=0,\;w_{A}=1.0,\;w_{ER}=0,\;w_{E}=0$
$opt_{ER}$	$w_{c}=0,\;w_{A}=0,\;w_{ER}=1.0,\;w_{E}=0$
$opt_{E}$	$w_{c}=0,\;w_{A}=0,\;w_{ER}=0,\;w_{E}=1.0$

## Data Availability

Data are contained within the article.
